# Spectral Data Processing for Field-Scale Soil Organic Carbon Monitoring

**DOI:** 10.3390/s24030849

**Published:** 2024-01-28

**Authors:** Javier Reyes, Mareike Ließ

**Affiliations:** 1 Department of Soil System Science, Helmholtz Centre for Environmental Research—UFZ, 06120 Halle, Germany; 2 Data Science Division, Department of Agriculture, Food, and Nutrition, University of Applied Sciences Weihenstephan-Triesdorf, 91746 Weidenbach, Germany

**Keywords:** carbon sequestration, modeling, long-term field experiment, pedometrics, spectral correction

## Abstract

Carbon sequestration in soils under agricultural use can contribute to climate change mitigation. Spatial–temporal soil organic carbon (SOC) monitoring requires more efficient data acquisition. This study aims to evaluate the potential of spectral on-the-go proximal measurements to serve these needs. The study was conducted as a long-term field experiment. SOC values ranged between 14 and 25 g kg^−1^ due to different fertilization treatments. Partial least squares regression models were built based on the spectral laboratory and field data collected with two spectrometers (site-specific and on-the-go). Correction of the field data based on the laboratory data was done by testing linear transformation, piecewise direct standardization, and external parameter orthogonalization (EPO). Different preprocessing methods were applied to extract the best possible information content from the sensor signal. The models were then thoroughly interpreted concerning spectral wavelength importance using regression coefficients and variable importance in projection scores. The detailed wavelength importance analysis disclosed the challenge of using soil spectroscopy for SOC monitoring. The use of different spectrometers under varying soil conditions revealed shifts in wavelength importance. Still, our findings on the use of on-the-go spectroscopy for spatial–temporal SOC monitoring are promising.

## 1. Introduction

Soil organic carbon (SOC) is one of the most studied soil properties in diverse disciplines such as agriculture, plant science, ecology, and environmental science. It is of particular interest in the study of agricultural systems as an indicator of soil quality. Furthermore, it plays an important role in the context of climate change mitigation. With appropriate agricultural soil management, CO_2_ soil emissions can be mitigated and the soil carbon sequestration increased as these soils are far from their storage capacity [1,2]. The initiative “4 per mile” launched at the Paris COP 21 Climate Change [3], aims to increase the global SOC stocks by 0.4 percent per year through agricultural practices to mitigate the atmospheric CO_2_ concentration derived from anthropogenic activities [4]. The potential of reaching the desired SOC stocks should be assessed by considering the feasibility and effectiveness of management practices [5,6]. For this reason, spatial–temporal monitoring of SOC in soils under agricultural use is needed. 

Long-term field experiments (LTEs) provide good insight into monitoring changes in SOC stocks with regards to soil management, its temporal variability, and the balance under different treatments. LTEs have been established for more than a century to evaluate the effect of different agricultural management on soil and crop characteristics which can only be observed in the long term [7]. An overview of German LTEs was provided in previous studies [8,9], which identified a total of 205 LTEs with a minimum duration of 20 years, of which 140 trials are still ongoing. Of these studies, 50 have a duration between 49 and 99 years, and three have lasted more than 100 years. Most of the LTEs correspond to arable field crops (168), and most of them were established for fertilization experiments. However, repeated sampling and conventional laboratory measurements of SOC on all LTE plots are expensive in terms of manpower and analysis costs.

Striving toward more cost and time-efficient SOC data acquisition, the use of visible–near infrared (VIS-NIR, 400–2500 nm) spectroscopy has increased over the last years [10,11,12,13]. SOC predictions in combination with spectral data are done through model building by different approaches such as machine learning methods [11] and partial least squares regression (PLSR), with the latter being one of the most applied methods due to its capacity to address multicollinearity and achieve dimensionality reduction [14,15]. The raw spectral data are affected by instrumental noise and baseline variations. Thus, it is necessary to apply preprocessing methods such as scatter correction and spectral derivatives, although there is no standardized procedure concerning soil spectra. Model interpretation, and hence the identification of SOC-specific wavelengths, can be achieved through different approaches, some of which are intrinsically included in the PLSR model, i.e., regression coefficients, loading weights, explained variance, etc. [16]. The interpretation of the recorded signal information about SOC is not trivial, as spectral absorption features are caused by the stretching and bending of structural molecule groups that are embedded in a complex soil matrix. Due to the soil complexity, an overlap of spectral response from organic and mineral compounds is observed [17]. Although fundamental features associated with SOC are found in the MID-Thermal range (2500–25,000 nm), weak overtones and combinations of fundamental vibrations due to the bending and stretching of NH, OH, and CH groups dominate the VIS-NIR range [17,18,19]. Different examples of important wavelengths associated with SOC in the VIS range (400–700 nm) [14,20,21], and in the NIR range (700–2500 nm [21,22,23,24]) were identified. It is known that some wavelengths will have more relevance in a regression model as they are associated with specific soil properties [16,25]. 

Several factors affect spectral measurements. Steps included in the measurement protocol such as instrument type, instrument setup, replicate measurements, sample preparation, and internal standard impact the accuracy and precision of the obtained data [26,27]. While it has not been profoundly studied, multiple instruments/scanning environments can have a significant effect on the soil spectra, and, consequently, on the modeling [28]. The uncertainty propagation of the spectral signal in PLSR models considering repetitions and preprocessing methods was studied previously [29]. While under laboratory conditions most aspects can be controlled or accounted for, field measurements pose additional challenges: On-the-go measurements do not allow for replicate measurements of the same sample. Additionally, the contact between soil and spectrometer might be lost while the spectrometer is pulled through the soil, and the spectra are recorded under varying soil moisture [5,23,30,31,32]. Other factors to consider are surface roughness, crop residuals and/or roots, incident light, soil texture, bulk density, and soil structure [5,33,34], which add more disturbance effects to the spectral signal. In the case of LTEs, an additional effect of chemical fertilizer could also influence the spectral signal [35] and thus on the model building as different amounts are applied to the plot treatments. To remove the effect of field conditions on the spectra, different methods such as direct standardization, piecewise direct standardization, external parameter orthogonalization, and orthogonal signal correction [36] could be used when measurements obtained under laboratory conditions are available.

This study aims to evaluate the ability of soil spectroscopy obtained from proximal on-the-go measurements to predict SOC since these data could provide a valuable source for spatio-temporal monitoring of SOC variation at the field scale. Two instruments with different spectral resolutions were used to collect data under laboratory and field (on-the-go below ground and site-specific above ground) conditions to evaluate if the prediction of on-the-go spectroscopy can be used for SOC monitoring, by maintaining an adequate performance compared with the controlled laboratory conditions. Different preprocessing methods were applied to obtain the best model performance and evaluate the consistency between devices. A data correction of field measurements based on laboratory data to improve the predictive model performance for SOC estimation is done by using three different methods. The model interpretation with regards to wavelength importance in PLSR models is performed with two indices: regression coefficients (RC) and variable importance in projection (VIP) scores, to analyze the consistency of models based on wavelengths associated with SOC from two devices and different laboratory and field conditions. Hypotheses: (1) The study hypothesizes that on-the-go spectroscopy measurements are expected to reliably predict soil organic carbon variation at the field scale, maintaining adequate performance compared to controlled laboratory conditions. (2) Different preprocessing methods are hypothesized to impact model performance, with effectiveness influenced by device spectral characteristics and diverse on-the-go measurement conditions. (3) Data correction techniques based on laboratory data are anticipated to significantly improve the accuracy of on-the-go field models for SOC estimation. (4) Wavelength importance analysis is hypothesized to reveal consistent SOC-associated patterns across devices and varying laboratory and field conditions.

## 2. Methods

### 2.1. Study Area

**Figure 1:**
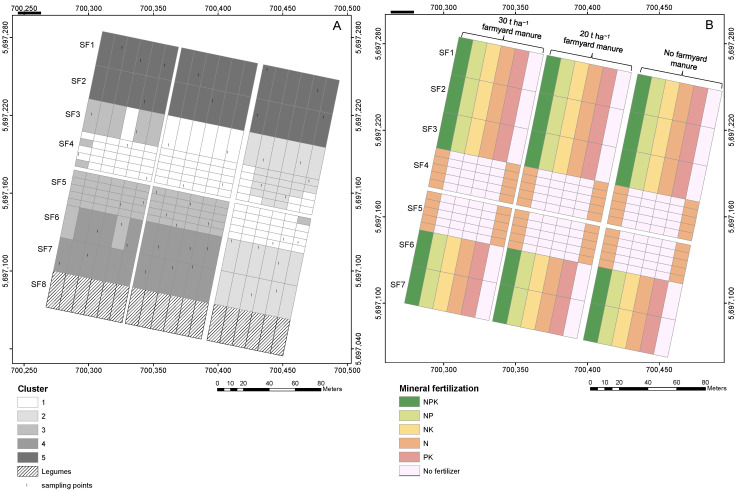
Study area located in Bad Lauchstädt. (**A**) Sampling point location; (**B**) fertilization treatments. Sampling points were selected by stratified random sampling. SF: subfield number. Coordinate reference system: EPSG 25833.

Data collection was conducted on the LTE site Static Fertilization Experiment in Bad Lauchstädt, Saxony-Anhalt, Germany (51°24’ N, 11°53’ E, 113 m a.s.l). The climate is characterized by an average total annual precipitation of 470–540 mm and an average mean temperature of 8.5–9.0 °C. The soil was classified as Haplic Chernozem developed from loess [37] according to the German soil classification system ([38]. Topsoil texture varies between highly clayey silt (Ut4) and highly silty clay (Tu4) according to the German soil survey system [38]. The Static Fertilization Experiment was initiated in 1902 by Schneidewind and Gröbler and is about 4 ha in size [39]. It has eight subfields (Figure 1A) and was initialized with a crop rotation of winter wheat—sugar beet—summer barley—potato. From 2015 onwards, sugar beet and potatoes were replaced by silage maize to reduce the workload. The crop rotation was initiated by different crops on adjacent fields so that all crops are always grown simultaneously on the experimental site. Subfield one is limed with 30 decitons every four years in spring. Since 1926, legumes were added to the crop rotation on subfield eight every seventh and eighth year. Additionally, the overall 288 plots differ according to their mineral and organic fertilizer treatments (Figure 1B). Farmyard manure is applied at 20 t ha^−1^ and 30 t ha^−1^, respectively, to one-third of the area of each field while the remaining third is left without organic fertilizer. Mineral fertilizer is applied in different combinations of N, P, and K, including the comparison of different N fertilizer types during a certain period. Subfields four and five of the experimental site were adapted in 1978 to investigate additional fertilizer treatments concerning different amounts of N in combination with an adapted organic fertilizer treatment. More details are given in [40]. The choice of the Static Fertilization Experiment LTE site was driven by the unique characteristics of this field. The site exhibits significant variability in soil organic carbon (SOC) content. This variability stems from the long-term fertilization treatment and crop rotation across its eight subfields.

### 2.2. Soil Organic Carbon Data

The soil samples were acquired at 50 locations, at 0–10 cm depth according to a stratified random sampling design (Figure 1A). Strata for random sampling were obtained by grouping the LTE plots according to their similarity by k-means cluster analysis. The following archive data were used to characterize each LTE plot: planted crops, agricultural treatment factors, total C, total N, available P, available K, and pH. In the end, 10 plots were randomly selected from each of the resulting 5 clusters, making a total of 50 plots to be sampled. Subsequently, one sampling point was randomly selected from each of the 50 plots excluding plot margins. The soil samples were air-dried, sieved (2 mm), and ground before carbon measurements with dry combustion. Total carbon was measured using the high-end elemental analyzer Vario EL cube CN (Elementar Analysensysteme GmbH, Langenselbold, Germany) with 3 replicates per sample. The carbonate content was also measured by the Vario EL cube CN, but values were below the detection level. Therefore, the total carbon was considered organic carbon.

### 2.3. Spectral Measurements

Spectral measurements were taken using two devices: ASD FieldSpec 4 Hi-Res by Malvern Panalytical, Malvern, United Kingdom, and Veris^®^ Vis-NIR spectrophotometer by Veris Technologies, Inc., Salina, USA (hereafter referred to as ASD and Veris, respectively). The ASD measures the Vis-NIR range (350–2500 nm), with a full-width half maximum of 3 nm in the Vis and 10 nm in the NIR, and an output of 1 nm spectral resolution. The Veris has an Ocean Optics USB4000 instrument (300 to 1100 nm) and a Hamamatsu TG series mini-spectrometer (1100 to 2200 nm), resulting in an output spectral resolution of 4–6 nm.

Field measurements were done with the ASD after crop harvest in sunny and dry soil conditions in September 2018. The spectra were measured at the soil surface at each sampling point using a 50 cm × 50 cm frame pointing north. Spectra were recorded at 5 replicate measurements with 3 external and 25 internal scans leading to a total of 15 spectra per sampling point. Veris field measurements were done the year after soil sampling in September 2019 due to logistic reasons. Several transects were recorded covering the entire field, with 2–3 m distance and a measurement depth of about 12 cm. The device is built in a shank pulled through the soil by a tractor; measurements are made through a sapphire window mounted on the bottom of the shank Approximately 20 spectra per second are acquired [41]. The volumetric water content was measured by using a TDR moisture sensor at the soil sampling locations, presenting values between 15–25% at the moment of acquiring on-the-go measurements. ASD and Veris laboratory measurements were made in air-dried and sieved (2 mm) samples. For these measurements, the Veris spectrometer was removed from the shank. Soil samples were divided into 3 subsamples and filled in Petri dishes. Each subsample was measured 3 times and rotated 90° to measure another 3 times resulting in 6 replicate measurements with 3 external scans each. Internally, the ASD was set to conduct 25 readings, and the Veris conducted 20 readings for each scan. Laboratory measurements resulted in 18 spectra per sample. 

### 2.4. Data Preparation for Model Building

In this study, SOC and spectral measurements were averaged for each sampling location. For Veris and ASD laboratory measurements, the 18 spectra per sample were averaged. For ASD field measurements the recorded 15 spectra per sampling point were averaged. From the Veris on-the-go field measurements, the 10 spectra nearest to each sampling point within the same LTE plot were averaged. The ASD spectra were affected by steps in reflectance values at the splice of the three sensors at 1000 and 1800 nm of the spectroradiometer. Consequently, an ASD splice correction was implemented using the *spectacles* R-package [42] which is based on the method described by [43]. For the Veris, the spectral range between 1000 to 1100 nm was removed due to the noise generated at the beginning and end of the two spectrometers. The spectral range selected for the model building was 400–2200 nm to allow for comparison between both devices, and to remove the beginning and end of the spectral range due to noise. Outliers were removed from the spectral measurements assigned to each sampling point by the adjusted quantile function in the *mvoutlier* package [44]. In the case of the Veris on-the-go data, the outlier removal was done before the selection of the 10 nearest spectra.

Data preprocessing aims to reduce the scattering effects that influence the spectral signal. There is no unique recommended preprocessing method to predict SOC. Therefore, different techniques were applied to observe the influence on model prediction and wavelength importance. The four applied combinations were: Savitzky–Golay (SG [45]), Savitzky–Golay + continuum removal (SGCR [46]), gap segment algorithm (gapDer [47]), and multiplicative scatter correction (MSC [48]). Details are presented in Table 1. SG, SGCR, and gapDer were obtained using the *prospectr* R-package [49] and the MSC by using the *pls* R-package [50]. A total of 16 datasets for the model building were obtained from a combination of the average spectral measurements using the two devices in the field and laboratory, and four different preprocessing techniques.

### 2.5. Model Building and Evaluation

PLSR [51] was applied to build regression models for SOC prediction. Model training, tuning, and evaluation were performed with a stratified 5-fold nested cross-validation (see details in [29]). To avoid spatial correlation between test and training data, neighboring samples within an 8 m distance were grouped in the same fold. Model evaluation was done with 5 repetitions. Thus, 25 PLSR models were obtained for each dataset. Equal data subdivisions were used to compare different preprocessing methods, spectrometers, and field versus laboratory measurements. Root mean square error (RMSE), R-squared, and relative percent difference (RPD) were used as error metrics of model performance. Also, the Concordance Correlation Coefficient [52] is presented in plots of predicted versus observed values.

### 2.6. Field Data Correction

**Figure 2:**
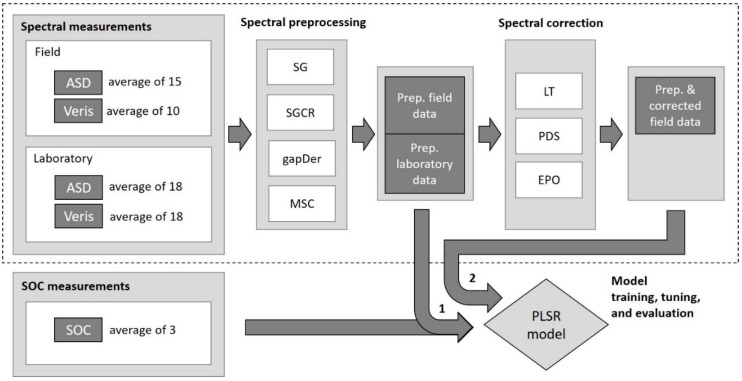
Procedure of PLSR model training, tuning, and evaluation based on the soil organic carbon and spectral data after spectral preprocessing only (1), and spectral preprocessing and correction (2). ASD: ASD FieldSpec 4 Hi-Res Spectrophotometer, Veris: Veris^®^ Vis–NIR spectrophotometer, SG: Savitzky–Golay, SGCR: Savitzky–Golay + continuum removal, gapDer: gap segment algorithm, MSC: multiplicative scatter correction, LT: linear transformation, PDS: Piecewise Direct Standardization, EPO: External Parameter Orthogonalization, and SOC: soil organic carbon content.

To improve the model performance of ASD and Veris field spectral data, three approaches were used for correction: linear transformation, piecewise direct standardization (PDS), and external parameter orthogonalization (EPO). The linear transformation was done by using a linear regression between the field and laboratory data of the average spectral value at each sampling location for each wavelength. The PDS algorithm [53] is a common method to relate each wavelength of a master spectrum with those of a secondary spectrum (laboratory and field in our case). The optimal parameters required to apply PDS are the PLSR number of components and the size of the wavelength window. This study considered a number of components of 1 to 10 and a window size of 1 to 20 for parameter tuning. The EPO [54] uses the projection of the primary and secondary data into an orthogonal space. The EPO algorithm components and the procedure for its calculation are described by [5]. It requires the determination of the number of EPO components. In our study, 1 to 10 EPO components were tested. To select the respective parameters for each data correction approach, nested cross-validation was applied following the same subdivision (external and internal validation) used for the PLSR model building. Figure 2 provides an overview of the procedure involving PLSR model training with the preprocessed spectral data (1) and the spectral data that underwent preprocessing and spectral correction (2). 

### 2.7. Wavelength Importance

Two indices were used to evaluate the wavelength importance in the PLSR models: RC and VIP. These indices can be obtained from the PLSR output and used for variable identification [55]. RCs are the coefficients associated with each wavelength to predict the response variables, and they are expected to have higher values (in absolute magnitude) when variables are important to the model prediction. The VIP scores [51] are calculated as the weighted sum of squares of the PLSR weights, which consider the amount of explained variance in each extracted latent variable. A common criterion used for VIP variable selection is to keep wavelengths with scores above 1 [56]. The equation is defined as:

(1) $${VIP}_{j}=\sqrt{\frac{p\sum_{a=1}^{A}\left[w_{aj}/{\left\|w_{a}\right\|}^{2}\right]}{\sum_{a=1}^{A\;}SS_{a}}}$$

where *p* is the total number of variables, *SS_a_* is the sum of squares explained by the *a*th PLSR component. Hence, the weights *w_j_* are a measure of the contribution of each variable according to the variance explained by each PLSR component where *w_aj_/‖w_a_*‖^2^ represents the loading weight (*w_a_*) importance of the *j*th variable. Meanwhile, the RCs were directly extracted from an object created using the *pls* package, and the VIP scores were calculated by using the *plsVarSel* R-package [55]. To compare the wavelength importance between different spectrometers and measurement conditions, local maxima and minima were identified for RC and the local maxima values for VIP, with a window width span of 100 nm for RC and 50 nm for VIP. This resulted in a lower number of wavelengths for the Veris data due to the comparatively lower spectral resolution. The plots of local peaks were done using the *ggplot2* R-package [57].

## 3. Results 

### 3.1. Performance Metrics

**Figure 3:**
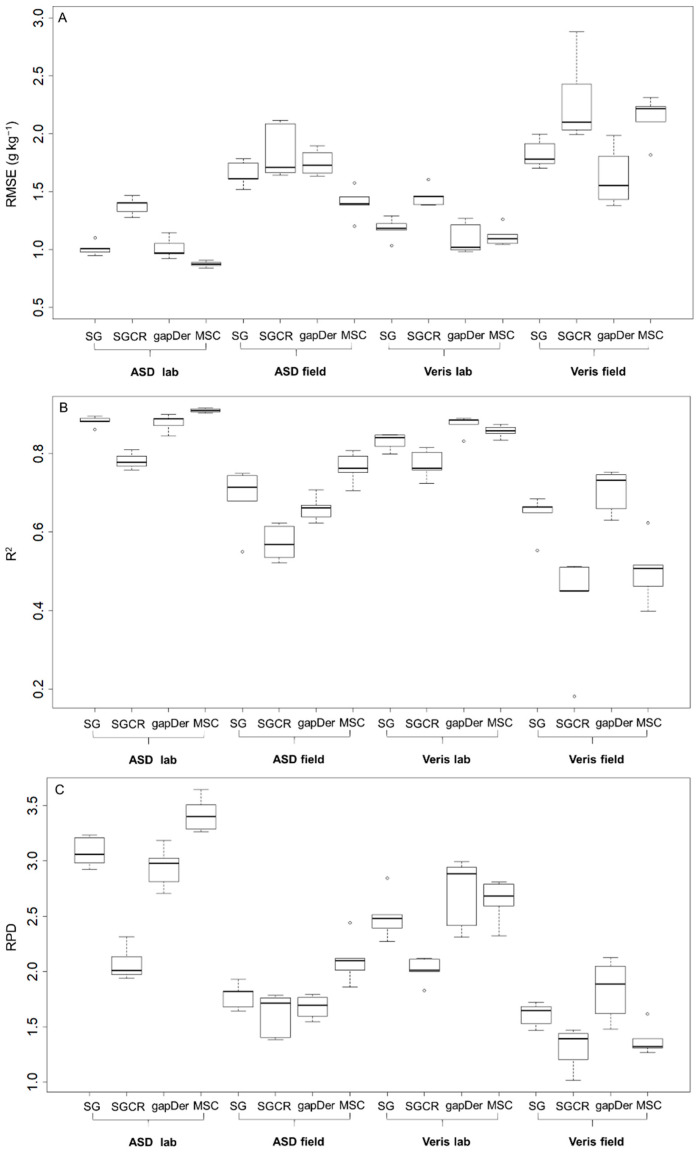
Predictive model performance of all 16 datasets (5 values per boxplot). (**A**) Root mean square error (RMSE), (**B**) R-squared (R^2^), and (**C**) Ratio of Performance to Deviation (RPD). SG: Savitzky–Golay, SGCR: Savitzky–Golay + continuum removal, gapDer: gap segment algorithm, MSC: multiplicative scatter correction. Boxes: interquartile range, whiskers: data range, circles: outliers, line: median.

**Figure 4:**
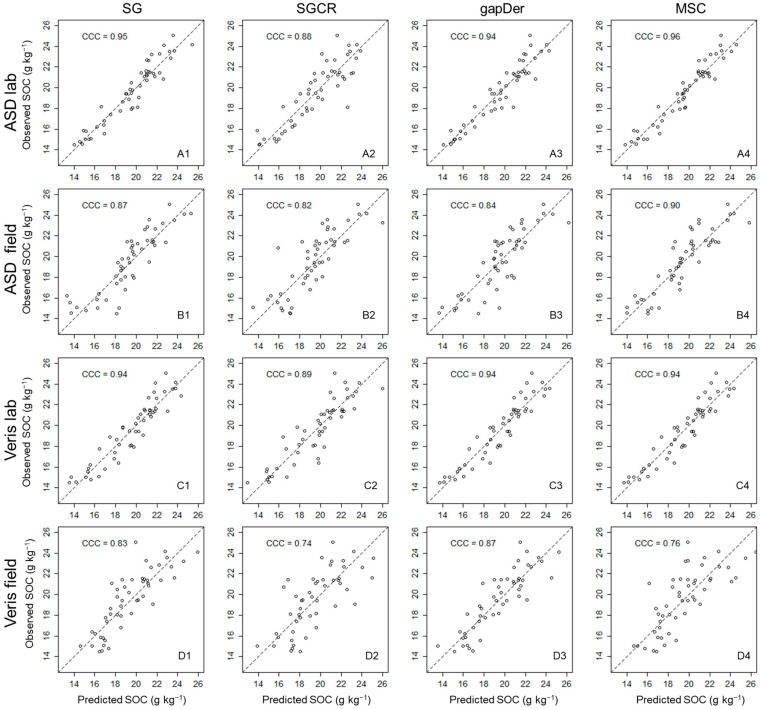
Predicted versus observed values for the 16 datasets (average of 5 predictions). CCC: concordance correlation coefficient. (**A**) ASD lab, (**B**) ASD field, (**C**), Veris lab, (**D**) Veris field, (**1**) SG: Savitzky–Golay, (**2**) SGCR: Savitzky–Golay + continuum removal, (**3**) gapDer: gap segment derivative, and (**4**) MSC: multiplicative scatter correction.

The measured SOC content has a mean value of 19.6 g kg^−1^ and a range of 14–25 g kg^−1^, showing the range of SOC values derived from the different fertilization treatments. Surprisingly, the carbonate content was below the detection limit even for the samples from subfield one, which is limed every four years. However, a possible reason might be that the grain size of the added lime might be larger than 2 mm and will, therefore, have been removed during sieving. Figure 3 presents the RMSE and R-squared boxplots from the five cross-validation repetitions for each dataset. For both devices, models built based on the laboratory measurements resulted in better predictive accuracy (Figure 3) and a lower dispersion with a higher concordance correlation coefficient in the 1:1 plots (Figure 4) in comparison with the field measurements. These results show the expected performance decline from ASD laboratory—Veris laboratory—ASD field—Veris field, even though the model performance of the Veris field data still shows adequate accuracy. The best model for each subgroup was: ASD laboratory–MSC (RMSE = 0.9 g kg^−1^, R^2^ = 0.9, RPD = 3.4), ASD field–MSC (RMSE = 1.4 g kg^−1^, R^2^ = 0.77, RPD = 2.1), Veris laboratory–gapDer (RMSE = 1.1 g kg^−1^, R^2^ = 0.86, RPD = 2.7), and Veris field–gapDer (RMSE = 1.6 g kg^−1^, R^2^ = 0.7, RPD = 1.8), showing that the best preprocessing method changed with the device but was maintained between laboratory and field when using the same spectrometer.

### 3.2. Corrected Field Data

**Figure 5:**
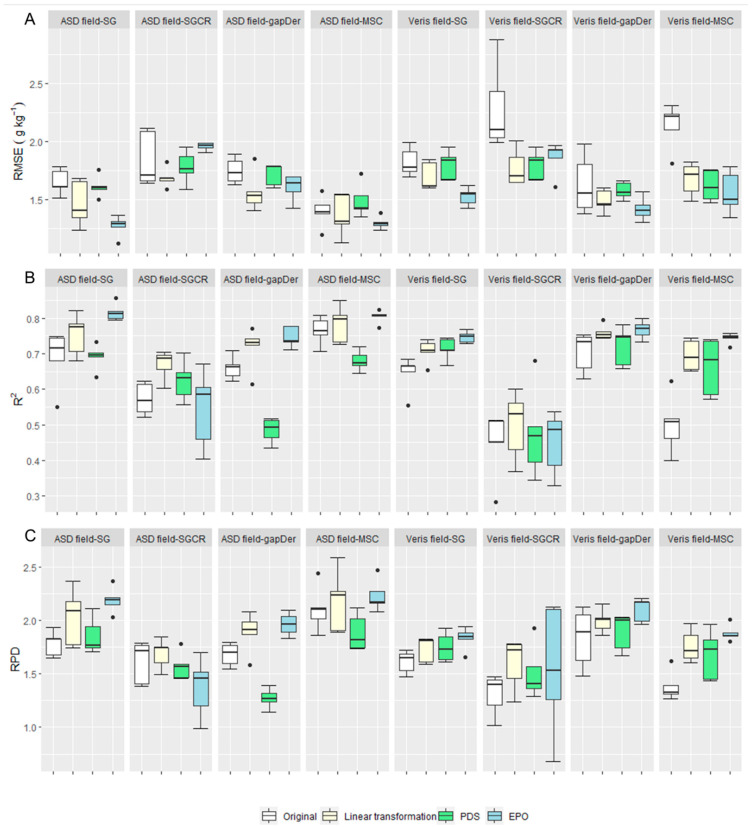
Predictive model performance of the 8 field datasets before and after spectral correction (5 values per boxplot). (**A**) Root mean square error (RMSE), (**B**) R squared (R^2^), and (**C**) Ratio of Performance to Deviation (RPD of the model prediction). SG: Savitzky–Golay, SGCR: Savitzky–Golay + continuum removal, gapDer: gap segment algorithm, MSC: multiplicative scatter correction, PDS: Piecewise Direct Standardization, EPO: External Parameter Orthogonalization. Boxes: interquartile range, whiskers: data range, circles: outliers, line: median.

**Figure 6:**
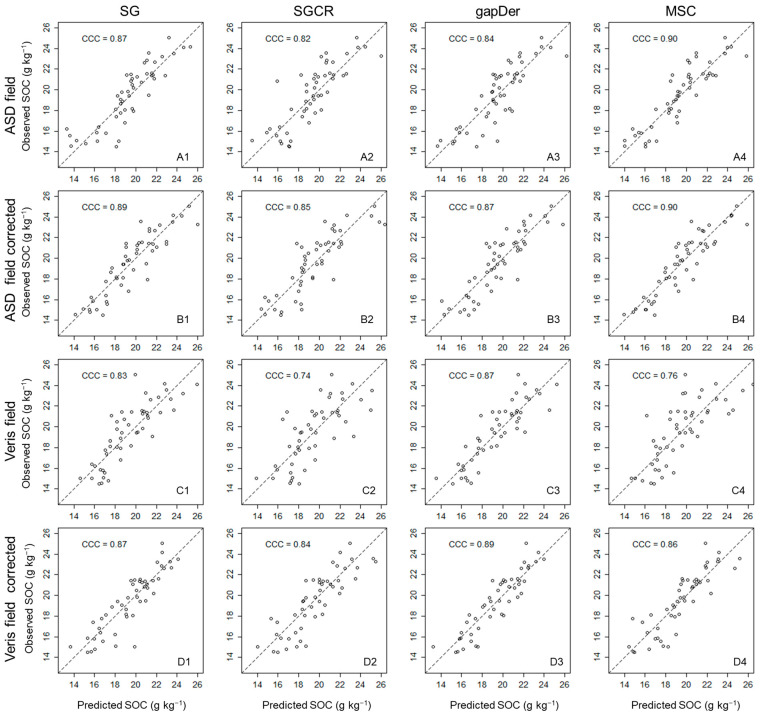
Predicted versus observed values comparing ASD and Veris field data before spectral correction and the best results after the data correction (average of 5 predictions). CCC: concordance correlation coefficient, (**A**) ASD field, (**B**) ASD field corrected, (**C**), Veris field, (**D**) Veris field corrected, (**1**) SG: Savitzky–Golay, (**2**) SGCR: Savitzky–Golay + continuum removal, (**3**) gapDer: gap segment derivative, and (**4**) MSC: multiplicative scatter correction.

To improve SOC estimation of models using field measurements with both ASD and Veris, corrections were done based on their corresponding laboratory data using three approaches: linear transformation, PDS, and EPO. The optimal parameter values for PDS and EPO are presented in Table 2. It can be observed that the optimal parameter values of Veris datasets for model building are lower compared with ASD for both the PDS and EPO algorithms. The predictive accuracy based on the field data was improved for both Veris and ASD data (Figure 5), although it was not equally effective among the preprocessing methods. In general, EPO was the best approach when comparing the different preprocessing methods apart from SGCR, where the best results were obtained with the linear transformation. Meanwhile, the performance of PDS was generally below the other two approaches. RPD values were >2 for the best models by using EPO in the ASD and Veris models in the field: ASD field–MSC (RMSE = 1.3 g kg^−1^, R^2^ = 0.80, RPD = 2.2) and Veris field–gapDer (RMSE = 1.4 g kg^−1^, R^2^ = 0.77, RPD = 2.1), respectively, which could be considered as an appropriate performance. Figure 6 displays the respective scatter plots; the corrected data tend to be less dispersed, and their concordance correlation coefficient is higher compared to the original field data. Nevertheless, even after correction, the predictive performance of the models trained on behalf of the corrected field data was still below that of the models trained with the laboratory data. By considering the highest improvement of the data correction in all cases, the comparison of the wavelength importance presented next is based on the spectrally corrected field data using EPO for SG, gapDer, and MSC, and linear transformation for SGCR preprocessing methods. 

### 3.3. Wavelength Importance 

**Figure 7:**
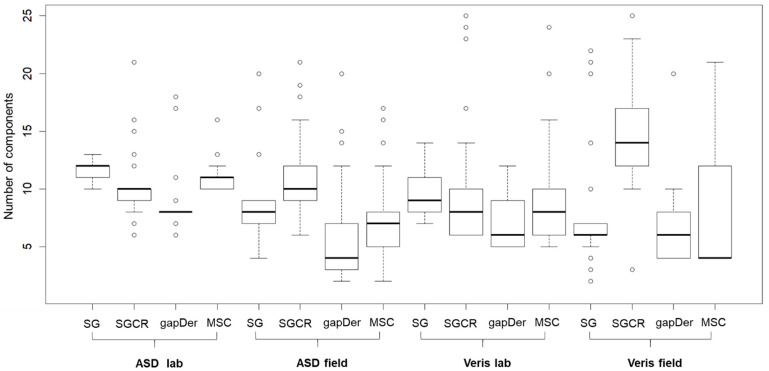
Boxplots of the tuned number of components of the 25 PLSR models built from each of the 16 datasets. SG: Savitzky–Golay, SGCR: Savitzky–Golay + continuum removal, gapDer: gap segment algorithm, MSC: multiplicative scatter correction. Boxes: interquartile range, whiskers: data range, circles: outliers, line: median.

Boxplots of the tuned number of PLSR components corresponding to the 25 models built for each of the 16 datasets are presented in Figure 7. The median value of the number of components was 15 or lower, and the variance of values for each of the 16 datasets differed according to the spectrometer, measurement condition, and preprocessing technique. In general, the models built with ASD and Veris laboratory data presented a lower dispersion compared with the respective models derived from field data. This corresponds to lesser disturbance effects, and, therefore, explains their better accuracy. Models derived after preprocessing with gapDer tend to have fewer components compared to the other preprocessing methods, which could be caused by the lower number of resulting variables when gapDer is applied to the raw data. 

**Figure 8:**
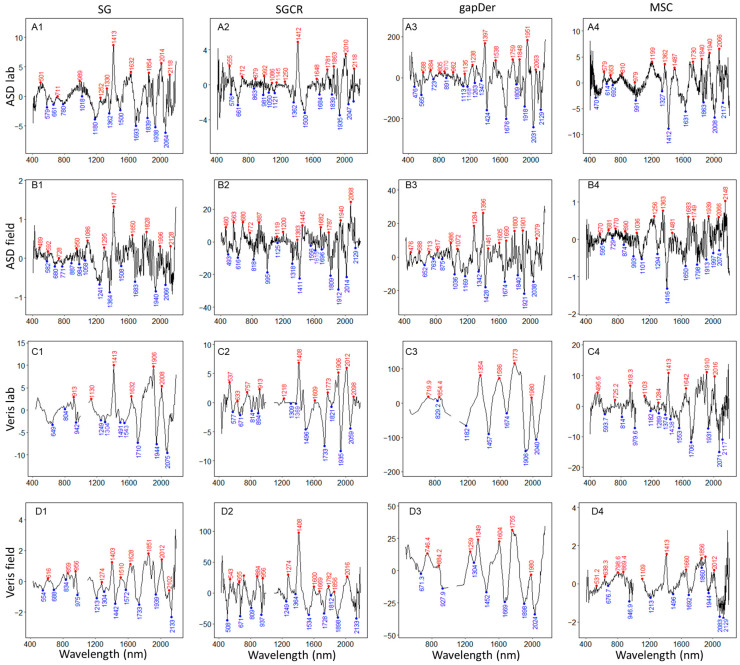
Median local maxima (red) and minima (blue) values of regression coefficients for each dataset. (**A**) ASD lab, (**B**) ASD field, (**C**), Veris lab, (**D**) Veris field, (**1**) SG: Savitzky–Golay, (**2**) SGCR: Savitzky–Golay + continuum removal, (**3**) gapDer: gap segment derivative, and (**4**) MSC: multiplicative scatter correction.

**Figure 9:**
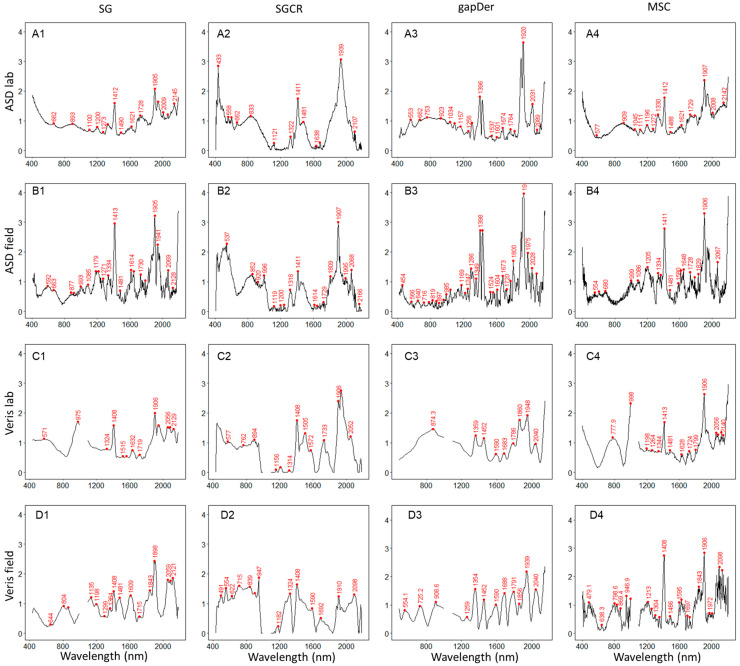
Median local maxima values of Variable Importance in Projection scores for each dataset. (**A**) ASD lab, (**B**) ASD field, (**C**), Veris lab, (**D**) Veris field, (**1**) SG: Savitzky–Golay, (**2**) SGCR: Savitzky–Golay + continuum removal, (**3**) gapDer: gap segment derivative, and (**4**) MSC: multiplicative scatter correction.

**Figure A1:**
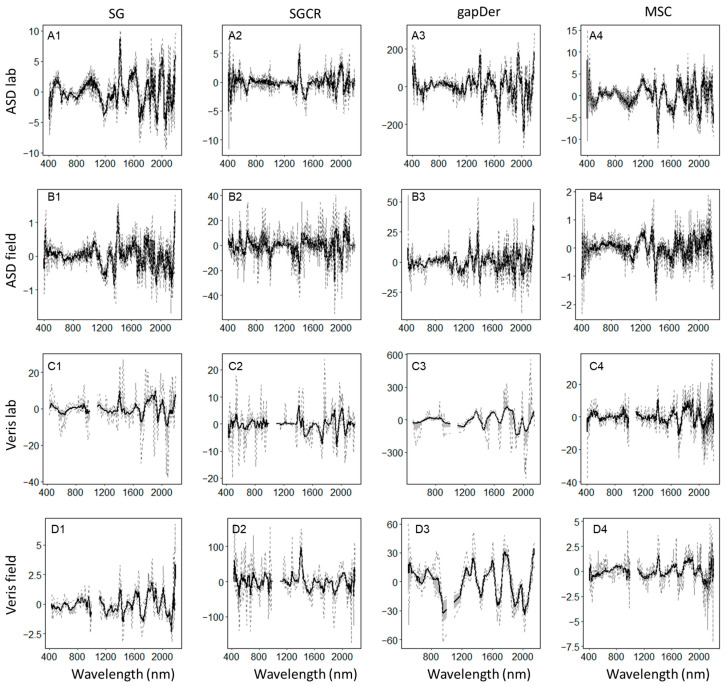
Regression coefficients from 25 models for each dataset. The black line is the median, the gray area shows the interquartile range, and the dashed lines represent the minimum and maximum values. (**A**) ASD lab, (**B**) ASD field, (**C**), Veris lab, (**D**) Veris field, (**1**) SG: Savitzky–Golay, (**2**) SGCR: Savitzky–Golay + continuum removal, (**3**) gapDer: gap segment derivative, and (**4**) MSC: multiplicative scatter correction.

**Figure A2:**
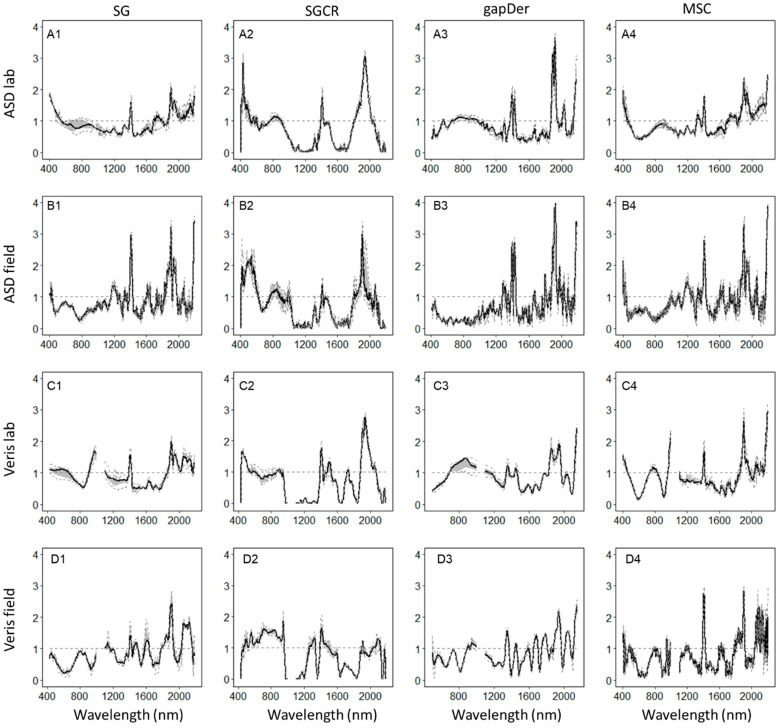
Variable Importance in Projection scores from 25 models for each dataset. The black line is the median, the gray area shows the interquartile range, and the dashed lines represent the minimum and maximum values. SG: Savitzky–Golay, SGCR: Savitzky–Golay + continuum removal, gapDer: gap segment algorithm, MSC: multiplicative scatter correction. Wavelengths above 1 (horizontal dashed line) could be considered as important for the models.

To facilitate the identification of wavelengths that relate to SOC and are therefore important independent of measurement condition, device, and preprocessing method, local peaks were identified for the median RC and VIP values (Figure 8 and Figure 9). More details about the dispersion and magnitudes of the RC and VIP values for each dataset are presented in Figure A1 and Figure A2, Appendix A. Table 3 presents the peak matches of VIP local maxima between the models obtained using ASD laboratory data with MSC preprocessing (best model performance) and those from the models built based on the other datasets. Due to differences in spectral resolution, the search range of peak matches was different for ASD (±10 nm) and Veris (±20 nm) datasets. For the RC local maxima and minima (Figure 8), several peaks were concentrated in the NIR range independent of the device or preprocessing method, and in some cases, there is a match in the local peaks between devices and methods, but the sign is flipped. Regarding VIP scores, some noticeable peaks were around 1400 and 1900 nm, and others were in the range of 1900–2200 nm. 

The local peaks of VIP scores with the data of best model performance (ASD laboratory using MSC), presented most matches with ASD laboratory data using SG preprocessing (11 matches), fewer matches with gapDer (7 matches), and fewest with SGCR (6 matches) following the order of model accuracy. Regarding the ASD field data, SG also presented 11 matches, followed by MSC (10 matches), SGCR (6 matches), and gapDer (4 matches). Concerning the comparison of Veris laboratory and field data with the best model, fewer matches were observed, Veris laboratory data presented most matches (9) with MSC preprocessing and 6–7 for all but the gapDer preprocessing which resulted in only 3 matches.

## 4. Discussion

### 4.1. Model Performance

The RMSE of the models is comparable to other studies when using ASD [58,59] and Veris [29] under laboratory conditions. It has better accuracy than the results observed by [41] with Veris on silty soils. The same applies to those based on ASD field measurements reported by [60,61], for sandy-loam and clay soils. An RPD value above 2 is considered to be acceptable when evaluating model performance. It was obtained for laboratory measurements with both devices. For ASD and Veris field measurements, it was reached with the best models after data correction, respectively. The lower accuracy of the models using field measurements is likely due to factors such as varying soil moisture, illumination, and surface roughness [58,62]. This is particularly evident with soil moisture, where studies have obtained better performance under dry conditions [5,63]. The difference in predictive model performance between ASD field and Veris field data is caused not only by the spectral resolution but also by the device-dependent characteristics in data acquisition. The Veris field data were collected on-the-go below ground. Accordingly, for model training to relate the spectral information to SOC, the average of spectral measurements close to the respective soil sampling location was derived. While the top centimeter was very dry during measurement, there was a notably higher soil moisture content at 12 cm depth. Additionally, in the on-the-go measurements, the soil contact of the sensor is affected during the movement due to the presence of clods and stones. Thus, the Veris field spectral measurements are affected by more disturbance effects compared to the site-specific above-ground ASD measurements. Surface roughness could also affect the model performance of the above-ground point ASD measurements due to the surface soil heterogeneity affecting the soil reflectance [33,64]. Models obtained from the ASD laboratory data showed better performance compared with the Veris laboratory data, displaying the effect of the inbuilt sensors and the lower spectral resolution. The soil spectral signal tends to have similar patterns; thus, small changes in the slope of the continuous data are better observed with the higher resolution of the ASD. Regarding differences among preprocessing techniques, MSC shows better results with models derived from ASD data, and gapDer with models derived from Veris data. SG consistently presented good performance in each device and preprocessing technique. MSC and SG are widely used [65,66]. MSC is a scatter-correction technique centered on reducing physical variability, thus facilitating the modeling of soil chemical effects. SG is a spectral-derivative that removes multiplicative and additive effects [67]. In the case of gapDer, it creates datasets with the lowest number of wavelengths due to the size of the smoothing window, resulting in a lower number of resulting variables. The one-year gap in Veris field measurements introduced a temporal discontinuity that may have implications for the study outcomes. Although unintended, such interruptions in data collection can introduce uncertainty into the final model outputs, potentially impacting the stability and robustness of predictions. Our analysis suggests that despite this temporal lapse, the overall predictive capacity of the models remains reasonable. However, acknowledging the importance of addressing such gaps in future studies is crucial, as they may impact the temporal dynamics of soil organic carbon. Future research endeavors should strive to minimize data gaps and systematically assess their implications on the accuracy and reliability of predictive models for soil organic carbon monitoring. 

### 4.2. Wavelength Importance

Differences in magnitude and sign of RC were identified, a finding which has also been reported by [28] when using different instruments. These differences may have been caused by models highly dependent on the instrument and scanning environment, hampering the transferability of models using different devices. The lower number of matching wavelength peaks in the models built based on the Veris data compared to the best model trained with ASD data was expected due to the lower information content of the spectra (lower spectral resolution). From the identified wavelengths that relate to SOC independent of the measurement condition, device, and preprocessing, the wavelength peaks around 1400 and 1900 are also related to other soil properties, particularly the stretching and bending of the O-H bonds of free water [68]. Regarding the importance of these wavelengths, [69] suggested that the retained water of air-dried soils influences SOC predictions because water retention increases with organic matter. Other high peaks in the range of 1900–2200 nm could be associated with the overtones and combination bands from CH compounds. More similarities in the local peaks were identified with VIP compared to RC. VIP demonstrated to be a good method to identify wavelength importance from local peaks; its usefulness has also been reported in other studies [59,70,71] concerning SOC prediction. In general, differences in wavelength importance were observed depending on the measurement condition, device, and preprocessing techniques, although with some concurrences at specific local peaks. It must be noted that both RC and VIP values are likely to change depending on the selected number of components in the PLSR (Figure 2), which in turn also depends on the respective preprocessing method and spectral resolution of the recorded data. 

Several of the wavelengths considered important for SOC prediction in both RC and VIP were found in the NIR range, agreeing with results reported by [70,72], who used a successive-projections algorithm and VIP scores, respectively. These results could be attributed to a stronger influence of CH bands, and water content in the case of ASD and Veris in the field, as it is observed on the peaks of RC values around 1400 nm and 1900 nm in both cases (Figure 9B,D). A notorious peak around 950–1000 nm of VIP values in the case of the Veris field datasets (Figure 8D), could be attributed to soil water, although some wavelengths in this range were also identified as important for SOC in other studies [25,73]. In contrast to our findings, other studies report a dominance of important wavelengths in the VIS range [74,75]. These differences could be attributed to the soil particularities [58,69,76], measurement settings (e.g., spectrometer, protocols), and preprocessing techniques since SOC influences a wide range of wavelengths in the Vis-NIR region. In line with our findings, the performance of the three independent spectrometers and their corresponding wavelength ranges included in an ASD field spectrometer and reported the 1800–2500 nm range was evaluated by [25] to result in better predictive performance for SOC. 

When comparing different preprocessing methods, patterns were more similar in the models derived from ASD measurements compared with those derived from Veris data. This is in line with our other results. The models built with differently preprocessed ASD laboratory data presented similar model performance (Figure 3) and low variation in the number of components (Figure 6). Preprocessing techniques can significantly affect both predictive performance [67] and wavelength importance [77]. The purpose of testing different processing methods in this work was to obtain the best possible result for the on-the-go measurements, which were found in this work to be different compared to the point above-ground ASD field measurements. The Veris data preprocessed with gapDer resulted in the best models for both laboratory and field data. This could be caused due to the total number of local peaks being lower with gapDer compared with the other preprocessing methods, and by differences in resolution, while it is more similar when the same preprocessing method is used (MSC). Matching patterns of important wavelengths independent of the preprocessing method are promising, as it implies that these wavelengths are ultimately reflecting the response of the soil characteristics rather than being caused by the data transformation. Some of the most frequent important wavelengths independent of the devices used in the field and laboratory and the preprocessing methods are like those reported by other studies: 560 nm [20], 1330 nm [78], 1400 nm, and 1900 nm [68], and 1720 nm [79]. 

### 4.3. Field Measurements and Data Correction for SOC Monitoring

The spectral correction of the field data on behalf of the laboratory data improved the model performance, which could help to obtain better information under field conditions. Concerning the parameters for PDS and EPO, lower values were determined for Veris compared to ASD which could be expected due to the lower spectral resolution of the Veris data. The best results were obtained by using the EPO method; meanwhile, linear transformation showed better results than the PDS algorithm when comparing several datasets, indicating that it is also a valid alternative for data correction. Nawar et al. [36] also found the best performance with EPO compared to PDS when using a cubist model for SOC prediction, which they related to the capacity of the EPO algorithm to remove the effect of soil moisture in the spectral signal. Nevertheless, the different data correction approaches satisfactorily improved SOC prediction based on the spectral field data. This is of particular importance in the context of spatially continuous SOC monitoring, which must be conducted under field conditions. However, while spectral data correction with site-specific spectral soil measurements is commonly applied to remote-sensing data [58,72,80,81], the spectral correction of proximal-sensed spectral field measurements with spectral laboratory data is less commonly used. A few examples related to SOC prediction employ PDS (e.g., [36,82]) and EPO [36,83,84], but applications related to on-the-go spectral recordings are scarce (e.g., [36]). Further work regarding the effects of environmental conditions in the on-to-go spectral measurements should be derived on building better models, as this factor could be quantified and used in the model building; nevertheless, the data correction in our study allowed us to reduce the impact on the models. Even if there is still a gap between laboratory and field results, our findings indicated a promising approach to be considered while using on-the-go spectral field measurements for SOC monitoring. Despite the loss of accuracy in SOC estimation using field measurements, it is possible to identify similarities in the wavelength importance between models from field and laboratory data, including the Veris on-the-go field measurements, which had been sampled at different depths and dates, indicating that even with additional uncertainties, the models can show relevant wavelengths associated with SOC. Another aspect to consider is that our experiment was developed in an LTE field that has a higher SOC variability compared to a conventional field with homogeneous soil management, which could lead to differences in both the model building and the overall SOC prediction. This consistency of the models is not only important for SOC monitoring with on-the-go proximal sensing but also when combining data from different devices, and measurement protocols to build universal models of soil spectroscopy and establish it as a measurement method. The collection of worldwide data in large spectral libraries follows this line of thought (e.g., [59,85,86]). Further and comprehensive work is necessary to explore the use of on-the-go spectral measurements under different local conditions, as the transferability of the models is uncertain. In our local study, we found differences in the model performance according to the laboratory and field conditions, use of devices with different spectral resolutions and methods of acquisition (above ground and on-the-go below ground), and the preprocessing methods.

## 5. Conclusions

The PLSR models presented good performance to predict SOC from on-the-go field measurements to allow for spatial–temporal SOC monitoring. We demonstrated that spectral correction of the sensor’s field data with its laboratory data resulted in an improvement in predictive model performance, particularly by using the EPO algorithm where the Veris field–gapDer presented the best results (RMSE = 1.4 g kg^−1^, R^2^ = 0.77, RPD = 2.1), which is, as far as we understand, the first example of combining laboratory and field Veris data to improve the model performance under field conditions. Hence, we consider spectral correction not only important while using remotely-sensed spectral data (as commonly applied), but also while using proximally-sensed data for spatial–temporal SOC monitoring under field conditions and suggest including it in any protocol for spectral field measurements. 

The detailed model insight and interpretation of important wavelengths with regards to SOC detected matches in important wavelengths independent of the sensor and measurement conditions, showing the capability of the models to detect important wavelengths even when the measurement conditions and acquisition methods differ. This consistency justifies the application of the methodology due to the physical importance of the SOC–spectra relationship. Nevertheless, this detailed analysis also disclosed the challenge of using soil spectroscopy for SOC monitoring. Differences in wavelength importance were observed depending on the measurement instruments and preprocessing methods, where the best performance changed for each device (MSC for ASD and gapDer for Veris), adding complexity to identifying relevant wavelengths. This is also a key aspect to consider when building large spectral libraries to generate universal spectral models to establish soil spectroscopy as a measurement method for SOC. Further work is needed to explain the differences between sensors and measurement conditions to develop best-practice protocols and standards for soil spectroscopy.

## Figures and Tables

**Table 1:** Preprocessing methods and corresponding wavelength ranges.

Preprocessing Method	ASD Wavelength Range	Veris Wavelength Range	Abbreviation
Savitzky–Golay	405–2195	432–2201	SG
Savitzky–Golay w = 11 and continuum removal	405–2195	432–2201	SGCR
Gap segment algorithm (w = 11, s = 10)	415–2185	408–2186	gapDer
Multiplicative scatter correction	400–2200	403–2201	MSC

w = window size, s = segment size.

**Table 2:** Optimal parameter values of PDS and EPO used for model building.

	Veris			ASD		
	PDS		EPO	PDS		EPO
	ncomp	w	ncomp	ncomp	w	ncomp
SG	1	2	7	4	5	9
SGCR	2	4	5	5	8	8
gapDer	1	2	5	3	3	8
MSC	1	2	6	2	9	9

SG: Savitzky–Golay, SGCR: Savitzky–Golay + continuum removal, gapDer: gap segment algorithm, MSC: multiplicative scatter correction, ncomp: number of components, w: window size, PDS: Piecewise Direct Standardization, EPO: External Parameter Orthogonalization.

**Table 3:** Comparison of wavelength local maxima peak matches (indicated by an x) between ASD laboratory MSC and close peaks (±10 nm for ASD and ±20 nm for Veris) from the other datasets regarding Variable Importance in Projection scores. SG: Savitzky–Golay, SGCR: Savitzky–Golay + continuum removal, gapDer: gap segment algorithm, MSC: multiplicative scatter correction.

ASD Lab MSC Wavelength	Veris Lab				Veris Field				ASD Lab			ASD Field			
	SG	SGCR	gapDer	MSC	SG	SGCR	gapDer	MSC	SG	SGCR	gapDer	SG	SGCR	gapDer	MSC
557		x				x	x			x	x			x	x
909						x	x					x			x
1045											x			x	x
1111									x	x		x	x		
1196				x	x	x		x	x		x	x	x		
1272								x	x			x			x
1330	x	x		x		x			x	x		x			
1412	x	x	x	x	x	x		x	x	x	x	x	x		x
1488		x		x	x			x	x	x					x
1621	x			x	x	x			x	x	x	x	x		
1729	x	x		x	x			x	x			x	x		x
1784			x	x			x		x		x				
1907	x	x	x	x	x	x		x			x	x	x	x	x
2008								x	x			x		x	x
2142	x			x					x			x			x
Total matches	6	6	3	9	6	7	3	7	11	6	7	11	6	4	10

## Data Availability

The data is available from Ließ, M., Reyes, J., 2024. SOCmonit | V120 LTE Vis-NIR spectral soil data. https://osf.io/7xyne/.
